# Estimated mortality due to seasonal influenza in southeast of Iran, 2006/2007 to 2011/2012 influenza seasons

**DOI:** 10.1111/irv.13061

**Published:** 2022-10-26

**Authors:** Razieh Khajehkazemi, Mohammad Reza Baneshi, Angela Danielle Iuliano, Katherine M. Roguski, Hamid Sharifi, Joseph Bresee, AliAkbar Haghdoost

**Affiliations:** ^1^ Modeling in Health Research Center, Institute for Futures Studies in Health Kerman University of Medical Sciences Kerman Iran; ^2^ Social Determinants of Health Research Center, Institute for Futures Studies in Health Kerman University of Medical Sciences Kerman Iran; ^3^ Influenza Division National Center for Immunization and Respiratory Diseases, Centers for Disease Control and Prevention Atlanta Georgia USA; ^4^ HIV/STI Surveillance Research Center, and WHO Collaborating Center for HIV Surveillance, Institute for Futures Studies in Health Kerman University of Medical Sciences Kerman Iran

**Keywords:** influenza, Iran, mortality, statistical model

## Abstract

**Background:**

Global estimates showed an estimate of up to 650,000 seasonal influenza‐associated respiratory deaths annually. However, the mortality rate of seasonal influenza is unknown for most countries in the WHO Eastern Mediterranean Region, including Iran. We aimed to estimate the excess mortality attributable to seasonal influenza in Kerman province, southeast Iran for the influenza seasons 2006/2007–2011/2012.

**Methods:**

We applied a Serfling model to the weekly total pneumonia and influenza (PI) mortality rate during winter to define the epidemic periods and to the weekly age‐specific PI, respiratory, circulatory, and all‐cause deaths during non‐epidemic periods to estimate baseline mortality. The excess mortality was calculated as the difference between observed and predicted mortality. Country estimates were obtained by multiplying the estimated annual excess death rates by the populations of Iran.

**Results:**

We estimated an annual average excess of 40 PI, 100 respiratory, 94 circulatory, and 306 all‐cause deaths attributable to seasonal influenza in Kerman; corresponding to annual rates of 1.4 (95% confidence interval [CI] 1.1–1.8) PI, 3.6 (95% CI 2.6–4.8) respiratory, 3.4 (95% CI 2.1–5.2) circulatory, and 11.0 (95% CI 7.3–15.6) all‐cause deaths per 100,000 population. Adults ≥75 years accounted for 56% and 53% of all excess respiratory and circulatory deaths, respectively. At country level, we would expect an annual of 1119 PI to 8792 all‐cause deaths attributable to seasonal influenza.

**Conclusions:**

Our findings help to define the mortality burden of seasonal influenza, most of which affects adults aged ≥75 years. This study supports influenza prevention and vaccination programs in older adults.

## 1 INTRODUCTION

Influenza is an acute viral respiratory infection that causes substantial mortality and morbidity every year.^1^ A recent global study estimated that an annual total of 290,000 to 650,000 respiratory deaths are attributable to seasonal influenza.^2^ However, these estimates are lacking in many developing countries.

Influenza infections are not routinely confirmed virologically; thus, they are infrequently recorded on death certificates.^3^ Moreover, virological testing might have been completed later in the course of illness, when complications from an influenza infection might be present and the virus is no longer detectable.^4^ Therefore, quantifying the seasonal burden of influenza is usually done by statistical modeling approaches.^5^ There are many types of time series regression models that have been used to estimate influenza‐associated excess mortality.^5^, ^6^, ^7^ Most require robust and reliable influenza virus surveillance data.^6^ Among them, the Serfling regression model^8^ requires the least amount of data, a minimum of 5 years with or without surveillance data.^6^ Therefore, it might be considered the best choice to estimate the influenza burden in data scarce situations.

The mortality attributed to seasonal influenza has been studied in several countries of WHO regions. For example, some of the countries in the African (South Africa^9^), Americas (Argentina^10^), South‐East Asia (Thailand^11^), European (Spain^12^), and Western Pacific regions (New Zealand^13^) have published estimates of influenza‐associated excess deaths. However, these estimates have not been made in countries of the WHO Eastern Mediterranean Region (EMR). Further, in Iran, there is little information regarding the burden of seasonal influenza. To our knowledge, this is the first study estimating the mortality impact of seasonal influenza in Iran. We aimed to estimate the annual influenza‐associated excess mortality in Kerman province for 2006/2007 to 2011/2012 influenza seasons by death category and age group and extrapolate our findings to the entire country.

## 2 METHODS

### 2.1 Study location

This study was done in Kerman province, southeast Iran. It is geographically the largest and demographically the ninth most populous of Iran's 31 provinces with a population of 3.16 million, of which 2.2% were aged ≥75 years^14^ (Text S1).

### 2.2 Mortality and population data

Individual mortality records for the period of March 21, 2006, to March 19, 2013 (corresponded to seven Iranian calendar years), were obtained from the death registry office at Ministry of Health and Medical Education (MOHME) of Iran. Text S1 included a brief information about the death certificate and death registry process in Iran. Each record included information on the date of birth and death, age (in years), and ICD‐10 code. Deaths due to stillbirth and those with unknown birthdate were removed. Data were aggregated into the weekly number of underlying pneumonia and influenza (PI; ICD‐10 codes J10‐J18), respiratory (ICD‐10 codes J00‐J99), circulatory (ICD‐10 codes I00‐I99), and all‐cause (ICD‐10 codes A00‐U99) deaths and stratified by three age groups (≤64, 65–74, and ≥75 years).

The age‐specific and total population estimates were obtained from the Statistical Center of Iran^14^ for censuses in 2006, 2011, and 2016. Weekly population estimates were interpolated by a linear regression model and merged with weekly mortality data.

### 2.3 Definition of influenza season

As Iran used the northern hemisphere formulation for seasonal influenza vaccine,^15^ the weeks used to define influenza seasons were July 1 (calendar Week 27) to June 30 (calendar Week 26) of the following year.^7^ The winter season, when the circulation of influenza viruses is greater, was defined to start on Week 40 of a year and end on Week 20 of the next year.^16^ We defined week, like the Iranian calendar, to start on Saturday and end on Friday. As the Iranian calendar starts 79 days (80 in leap years) later than Gregorian calendar, only those weeks which made a full influenza season were included in the analyses. So, the study period was comprised of six complete influenza seasons, from 2006/2007 to 2011/2012.

### 2.4 Definition of influenza epidemic periods

As we did not have robust viral surveillance data, we used PI death rates in winter as a proxy for influenza activity.^7^, ^8^

**FIGURE 1:**
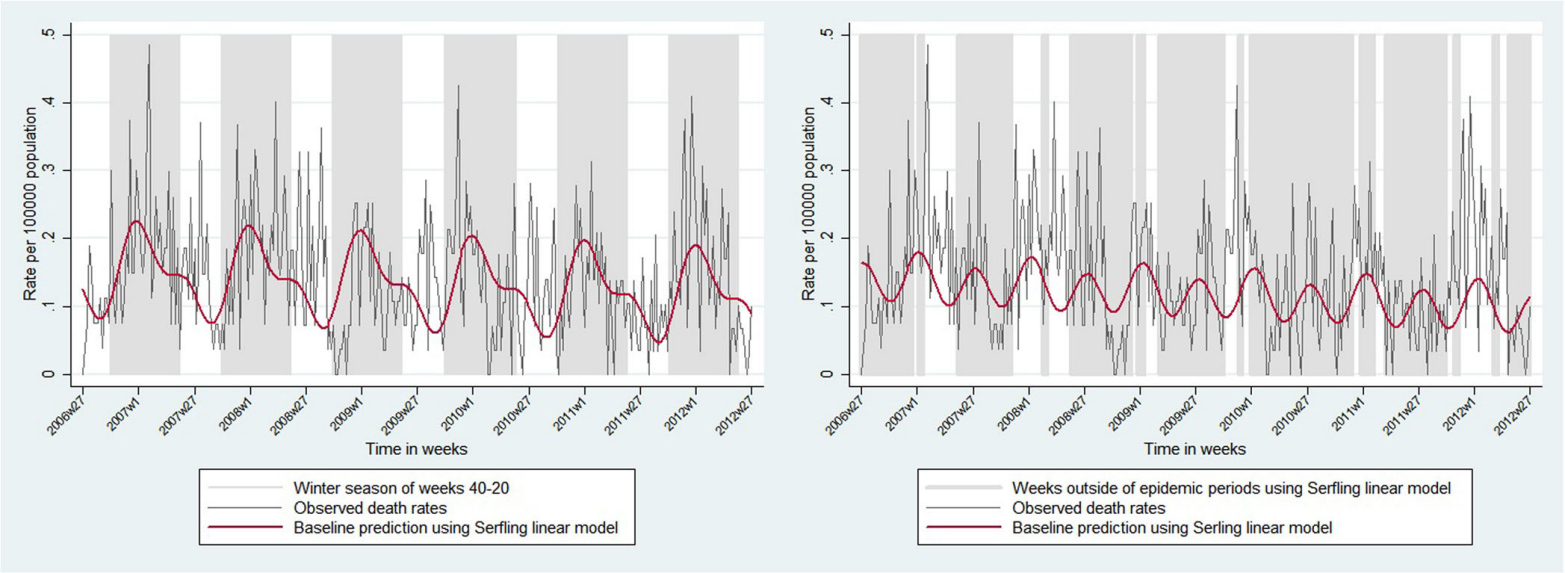
Time periods used in Serfling models to define epidemic periods and estimate baseline mortality rates. The observed weekly total pneumonia and influenza (PI) death rates per 100,000 population (depicted by thin gray line) along with the time periods used in each Serfling linear regression model (depicted by gray areas) and the fitted baseline prediction line (depicted by thick dark red line). The left panel shows the time periods (i.e., winter seasons, Week 40 to Week 20 of the next year) of total PI death rates per 100,000 population were used to define the epidemic periods and the fitted baseline prediction line. The right panel shows the time periods (i.e., weeks outside of epidemic periods) of total PI death rates per 100,000 population were used to estimate the seasonal baseline mortality rate in the absence of influenza and the fitted baseline prediction line.

We fitted Serfling linear regression model on observed weekly total PI death rates after excluding summer Weeks 20–40^7^ (Figure 1). The model included linear time trend variable and trigonometric functions to adjust for seasonal fluctuations in data:

$$y_{t}=\alpha +\beta_{1}t+\beta_{2}\sin\left(\frac{2\mathrm{\pi t}}{52.17}\right)+\beta_{3}\cos\left(\frac{2\mathrm{\pi t}}{52.17}\right)+\beta_{4}\sin\left(\frac{4\mathrm{\pi t}}{52.17}\right)+\beta_{5}\cos\left(\frac{4\mathrm{\pi t}}{52.17}\right)+\varepsilon_{t},$$

where $y_{t}$ is the weekly total PI death rates per 100,000 population in week *t*, *t* is the week number, *α* is the intercept, $\beta_{1}$ is the coefficient for time trend, $\beta_{2}$ to $\beta_{5}$ are coefficients for annual and semi‐annual seasonal fluctuations in data, and $\varepsilon_{t}$ is the normally distributed error terms.

Epidemic weeks were defined as any week during winter season where the observed PI death rate exceeded the one‐sided upper 95% confidence interval (CI) of the baseline prediction model.^7^, ^8^ The start of each epidemic period was defined either by observing ≥2 consecutive epidemic weeks and/or one epidemic week followed by one non‐epidemic week followed by one another epidemic week, to be more conservative; and the epidemic period ended with ≥3 consecutive non‐epidemic weeks.^7^ Therefore, in each season ≥1 epidemic periods were possible.

### 2.5 Estimation of influenza‐associated deaths

We applied Serfling model to the interrupted time series of age and death cause groups, after excluding weeks inside the epidemic periods, to predict the weekly baseline mortality rates and 95% CIs in the absence of influenza activity^7^ (Figure 1). Then, we calculated the number of predicted deaths for each week inside the epidemic periods, which is the expected number of deaths in the absence of influenza, and its 95% CI. Observed deaths were the underlying PI, respiratory, circulatory, and all‐cause deaths obtained from the death registry. Weekly excess deaths were calculated as the observed minus predicted deaths and their lower and upper limits were calculated by subtracting the upper and lower CIs of weekly baseline predicted deaths from observed deaths, respectively. Negative values of weekly excess deaths were replaced with zero,^17^ because we assumed that influenza did not have a protective effect on death. We summed the weekly excess deaths in each season to calculate the seasonal excess mortality and used population estimates by age group to calculate rates per 100,000 population; this approach was used to calculate the upper and lower 95% confidence limits of seasonal excess mortality.^18^ The annual excess mean number and rate were calculated across all influenza seasons.

Due to the excess zeros in the age‐specific PI mortality data, only the total influenza‐associated PI mortality were calculated.

As Kerman is the province most representative of Iran's climate^19^ and has nearly the same proportion of people aged ≥75 years as the whole country (2.2% vs. 2.5%),^14^ to have a general overview of the influenza‐associated deaths for the country, we applied the mean of age‐specific and total annual excess death rates estimated for Kerman province across the six seasons to the age‐specific and total population of Iran at the time of 2016 census.^14^

### 2.6 Over‐dispersion assessment

To assess the impact of over‐dispersion in our data, we applied the Serfling approach in the negative binomial regression model,^20^ both to define the influenza epidemic periods and estimate the excess deaths associated with influenza. Our Serfling negative binomial model was

$$y_{t}=\begin{array}{l} \alpha *\exp(\beta_{1}t+\beta_{2}\sin\left(\frac{2\mathrm{\pi t}}{52.17}\right)+\beta_{3}\cos\left(\frac{2\mathrm{\pi t}}{52.17}\right)+\beta_{4}\sin\left(\frac{4\mathrm{\pi t}}{52.17}\right)\\ +\;\beta_{5}\cos\left(\frac{4\mathrm{\pi t}}{52.17}\right))\delta_{t}, \end{array}$$

where $y_{t}$ is the weekly number of deaths in each age group and death cause category, *α* is the offset term (log of weekly age‐specific population), and $\delta_{t}$ is the over‐dispersion parameter. Other terms were similar to the Serfling linear model.

All analyses were done with Stata (Version 13).

## 3 RESULTS

### 3.1 Overall mortality

A total of 78,454 all‐cause deaths occurred in Kerman province from March 21, 2006, to March 20, 2013. There were 1331 deaths with stillbirth diagnosis, 96 with an unknown birthdate, and 11,360 occurred before Week 26 of 2006 or after Week 27 of 2012.

Across the influenza seasons 2006/2007–2011/2012, 1252 deaths were coded as underlying PI, 6704 as respiratory, and 23,833 as circulatory diseases. The mean (range among years) annual mortality rate per 100,000 population was 7.5 (6.4–9.0) for PI, 40.0 (36.5–41.6) for respiratory, 142.0 (128.8–153.3) for circulatory, and 391.5 (367.6 to 409.3) for all‐cause deaths. In most years, adults ≥75 years represented more than 50% of deaths due to underlying PI, respiratory, and circulatory diseases (Table S1).

### 3.2 Estimates of influenza‐associated mortality

Table 1 shows the estimated mean excess death number and rate per 100,000 population attributed to seasonal influenza by age groups and cause of death categories for Kerman province and the extrapolated figures for Iran. The mean annual influenza‐associated PI mortality rate per 100,000 population was 1.4 (95% CI 1.1–1.8), which corresponds to an annual mean of 40 (95% CI 31–50) excess PI deaths in Kerman province. We estimated that 242 excess PI deaths occurred during the study period in Kerman province, which is 19.3% (242/1252) of all underlying PI deaths in this region.

**TABLE 1:** Estimated influenza‐associated mortality by age groups and underlying cause of death, influenza seasons 2006/2007–2011/2012

Underlying cause of death	Age group	Total excess death numbers in Kerman province^a^	Mean annual excess death number in Kerman province (95% CI)	Mean annual excess death rate per 100,000 population in Kerman province (95% CI)	Mean annual excess number of deaths in Iran^b^ (95% CI)
PI	≤64	NA	NA	NA	NA
	65–74	NA	NA	NA	NA
	≥75	NA	NA	NA	NA
	Total	242	40 (31–50)	1.4 (1.1–1.8)	1119 (879–1439)
Respiratory	≤64	149	25 (17–35)	0.9 (0.6–1.3)	675 (450–976)
	65–74	112	19 (13–26)	24.0 (17.3–33.1)	693 (500–956)
	≥75	339	56 (43–73)	100.2 (76.3–128.7)	1986 (1513–2551)
	Total	600	100 (73–134)	3.6 (2.6–4.8)	2877 (2078–3836)
Circulatory	≤64	123	21 (13–35)	0.8 (0.5–1.3)	600 (375–976)
	65–74	140	23 (14–38)	30.2 (17.9–48.2)	873 (517–1393)
	≥75	300	50 (33–73)	90.2 (58.9–130.5)	1788 (1168–2587)
	Total	563	94 (60–146)	3.4 (2.1–5.2)	2717 (1678–4156)
All cause	≤64	812	135 (88–198)	5.1 (3.3–7.4)	3828 (2477–5554)
	65–74	302	50 (33–72)	64.2 (43.2–92.1)	1855 (1248–2661)
	≥75	726	121 (83–166)	215.2 (146.7–295.7)	4266 (2908–5862)
	Total	1840	306 (204–436)	11.0 (7.3–15.6)	8792 (5835–12,468)

Abbreviations: CI, confidence interval; NA, not applicable (due to excess zeros in the age‐specific PI mortality data); PI, pneumonia and influenza.

^a^ Calculated by sum of excess death numbers in each influenza seasons from Table 2.

^b^ Estimates for Iran were calculated by applying excess death rates per 100,000 population in Kerman province to the age‐specific and total population of the country.

The average annual rate of influenza‐associated mortality per 100,000 population was 3.6 (95% CI 2.6–4.8) and 3.4 (95% CI 2.1–5.2) for underlying respiratory and circulatory deaths, respectively. These corresponded to an annual mean of 100 (95% CI 73–134) excess respiratory and 94 (95% CI 60–146) excess circulatory deaths in Kerman province. We estimated that 8.9% (600/6704) and 2.4% (563/23,833) of all respiratory and circulatory deaths were attributable to seasonal influenza, respectively. Adults aged ≥75 years accounted for 56.0% (56/100) and 53.2% (50/94) of all respiratory and circulatory excess deaths, respectively. The age‐specific influenza‐associated respiratory and circulatory mortality rate varied by age group. It was highest among people aged ≥75 years and lowest among ≤64 years. People aged ≥75 years were four times and three times more likely to die from influenza‐associated respiratory and circulatory causes, respectively, compared to people aged 65–74 years.

For all‐cause deaths, the mean annual excess mortality rates associated with influenza was 11.0 (95% CI 7.3–15.6) per 100,000 population. We estimated that an annual mean of 306 (95% CI 204–436) deaths were associated with influenza in Kerman province, which is 2.8% (1840/65,667) of all‐cause deaths occurred during the study period in this region.

Extrapolation of our findings to the country showed that in each influenza season a total of 1119 (95% CI 879–1439) excess underlying PI, 2877 (95% CI 2078–3836) respiratory, 2717 (95% CI 1678–4156) circulatory, and 8792 (95% CI 5835–12,468) all‐cause deaths would occur. Adults ≥75 would account for 69% and 65% of all influenza‐associated respiratory and circulatory deaths in Iran.

### 3.3 Estimates of influenza‐associated mortality by influenza season

The excess mortality associated with influenza varied by influenza seasons. The highest PI and respiratory excess death rates per 100,000 population were observed in the 2011/2012 influenza season followed by the 2007/2008 season. However, the highest circulatory and all‐cause excess death rates per 100,000 population were observed in the 2007/2008 season (Table 2).

**TABLE 2:** Estimated influenza‐associated mortality by age groups, underlying cause of death, and influenza seasons 2006/2007–2011/2012, Kerman province, Iran

Underlying cause of death and seasons	Excess death number				Excess death rate per 100,000 population (95% CI)			
	≤64	65–74	≥75	Total	≤64	65–74	≥75	Total
PI								
2006/2007	NA	NA	NA	44	NA	NA	NA	1.6 (1.3–2.1)
2007/2008	NA	NA	NA	61	NA	NA	NA	2.2 (1.7–2.8)
2008/2009	NA	NA	NA	12	NA	NA	NA	0.5 (0.3–0.6)
2009/2010	NA	NA	NA	35	NA	NA	NA	1.3 (1.0–1.5)
2010/2011	NA	NA	NA	17	NA	NA	NA	0.6 (0.4–0.8)
2011/2012	NA	NA	NA	73	NA	NA	NA	2.5 (2.0–3.0)
Respiratory								
2006/2007	38	38	48	124	1.5 (1.0–2.1)	50.6 (37.8–65.9)	94.9 (67.3–125.6)	4.6 (3.2–6.2)
2007/2008	33	24	86	143	1.3 (0.7–1.9)	31.6 (22.2–45.4)	163.7 (131.2–208.6)	5.3 (3.9–7.1)
2008/2009	16	4	20	40	0.6 (0.4–0.8)	5.3 (4.3–6.2)	36.6 (29.4–45.8)	1.4 (1.1–1.8)
2009/2010	19	11	18	48	0.7 (0.6–0.9)	14.0 (10.7–18.1)	32.0 (19.7–45.6)	1.7 (1.2–2.3)
2010/2011	10	4	21	35	0.4 (0.3–0.6)	5.3 (2.2–9.3)	35.0 (22.4–52.2)	1.2 (0.8–1.9)
2011/2012	33	31	146	210	1.2 (0.8–1.6)	37.5 (26.5–53.7)	239.2 (187.9–294.5)	7.2 (5.5–9.2)
Circulatory								
2006/2007	28	16	31	75	1.1 (0.5–2.0)	21.4 (8.2–37.0)	61.6 (25.4–98.8)	2.8 (1.2–4.9)
2007/2008	25	53	112	190	1.0 (0.6–1.7)	69.6 (49.2–97.4)	211.2 (160.0–273.5)	7.0 (5.1–9.7)
2008/2009	2	13	24	39	0.1 (0.0–0.3)	17.1 (7.3–31.3)	43.6 (25.8–64.9)	1.4 (0.7–2.5)
2009/2010	42	16	41	99	1.6 (1.3–1.9)	20.8 (12.3–31.6)	72.3 (54.1–98.5)	3.5 (2.7–4.7)
2010/2011	13	8	29	50	0.5 (0.3–0.8)	10.5 (4.5–17.6)	49.1 (27.6–79.6)	1.7 (0.9–2.9)
2011/2012	13	34	63	110	0.5 (0.1–1.2)	41.8 (26.4–74.5)	103.3 (60.9–167.8)	3.8 (2.1–6.8)
All cause								
2006/2007	92	40	69	201	3.6 (2.0–6.5)	53.6 (26.9–94.2)	134.9 (54.9–232.5)	7.5 (3.7–13.3)
2007/2008	295	96	242	633	11.3 (8.7–14.3)	126.0 (94.4–163.9)	457.5 (361.6–560.1)	23.3 (18.129.3)
2008/2009	77	36	60	173	2.9 (1.8–4.4)	46.8 (36.9–58.9)	108.6 (66.5–156.7)	6.2 (4.1–9.0)
2009/2010	70	28	36	134	2.6 (1.7–3.9)	34.8 (22.9–48.6)	63.9 (47.1–96.9)	4.8 (3.2–7.0)
2010/2011	91	16	70	177	3.3 (1.9–5.1)	20.1 (13.3–30.6)	118.6 (77.7–172.2)	6.2 (3.8–9.3)
2011/2012	187	86	249	522	6.7 (3.8–10.2)	103.9 (64.8–156.2)	407.9 (272.1–556.0)	17.9 (11.5–25.8)

Abbreviations: CI, confidence interval; NA, not applicable (due to excess zeros in the age‐specific PI mortality data); PI, pneumonia and influenza.

### 3.4 Over‐dispersion assessment

A total of 69 epidemic weeks were identified using Serfling linear and 67 using Serfling negative binomial models. The Spearman rank correlation coefficient was 0.97 (*p* value <0.0001) between epidemic weeks identified by these models. These two models showed the same duration of epidemic periods in all but the 2006/2007 season (Table S2). The estimates of influenza‐associated excess mortality using Serfling negative binomial model were consistent with Serfling linear model (Pearson correlation coefficient = 0.99 between total seasonal excess deaths estimated by the two models in each death cause category). The annual mean of PI, respiratory, circulatory, and all‐cause influenza‐associated excess mortality rates were 1.4 (95% CI 1.1–1.8), 3.4 (95% CI 2.5–4.7), 3.4 (95% CI 2.1–5.2), and 11.1 (95% CI 7.5–15.8) per 100,000 population, respectively (Table S3). The age‐specific estimates were also very similar to those obtained from Serfling linear model. The observed weekly mortality rates and baseline predicted rates per 100,000 population obtained by Serfling linear and negative binomial models among people aged ≥75 years are represented in Figures S1–S3 separately for respiratory, circulatory, and all‐cause deaths.

## 4 DISCUSSION

Our study, which is the first to estimate the mortality burden of seasonal influenza in Iran, may serve as a preliminary step toward understanding the epidemiology of influenza in the country and designing public health prevention strategies. We estimated that seasonal influenza was responsible for an annual mean of 40 (PI) to 306 (all cause) excess deaths in Kerman province during of the winter influenza seasons of 2006/2007–2011/2012. The lower and upper bounds of this range (40 to 306) correspond to an annual rate of 1.4 (95% CI 1.1–1.8) excess PI deaths and 11.0 (95% CI 7.3–15.6) excess all‐cause deaths per 100,000 population. If we assumed that Kerman province was representative of the general population of Iran (79.9 million in 2016 census^14^) and had the same circulation pattern of seasonal influenza viruses, we would expect 1119 (PI) to 8792 (all cause) deaths attributed to seasonal influenza across the country in each influenza season.

Our estimated PI excess death rate per 100,000 population is comparable with the estimates of 2.2 in Thailand,^11^ 1.4 in Brazil,^21^ and 2.9 in Singapore^22^ but is lower than 6.0 in Argentina,^10^ 4.1 in Hong Kong,^23^ and 3.7 in the United States.^24^ On the other hand, we attributed 19.3% of all observed PI deaths during the study period to seasonal influenza, which is higher than studies from the United States^24^ and Hong Kong^23^ (5% and 7.4%, respectively). This might be due to the inclusion of viral activity terms in the statistical models of these studies, the way excess deaths were calculated, the definition of epidemic periods or ICD codes used to define the death categories. Among EMR countries, a study in Oman^25^ used in‐hospital deaths and estimated an annual influenza‐associated death rate of 0.9 (95% CI 0.7–1.0), lower than our estimate using registered PI deaths.

The impact of influenza on the excess all‐cause mortality rate was similar to other studies done in China,^26^ Canada,^27^ and Hong Kong.^23^ Similar to China^26^ and Hong Kong,^23^ we observed 2.8% of all observed deaths were associated with influenza.

Our findings on the respiratory deaths attributed to influenza are consistent with estimates of China^26^ and Thailand^11^ but slightly lower than the United States.^24^ Additionally, we estimated a higher rate of influenza‐associated respiratory deaths, both in total and by age groups, than the global study which provided an estimate for Iran's influenza‐associated respiratory deaths through extrapolation simulation methods^2^; this might be due to the use of WHO global health estimates of respiratory infection mortality rates to categorize countries in analytic divisions instead of the country‐specific seasonal influenza case fatality ratio. Moreover, a constant seasonal influenza attack rate between countries was assumed. However, the ranges for the simulated estimates were broader. We estimated an annual excess rate of 0.9 (95% CI 0.6–1.3), 24.0 (95% CI 17.3–33.1), 100.2 (95% CI 76.3–128.7), and 3.6 (95% CI 2.6–4.8) per 100,000 population for people aged ≤64, 65–74, ≥75, and all ages, respectively, whereas the simulated Iranian estimates were 0.7 (95% credible interval [CrI] 0.2–1.6), 7.6 (95% CrI 1.3–26), 58.7 (95% CrI 8.2–150.1), and 2.2 (95% CrI 0.9–4.0) per 100,000 population, respectively. Nevertheless, we observed almost complete overlap between our CIs with those credible intervals calculated in the global study. This shows the need for more estimates from representative countries around the world, including Iran, in these global efforts to better estimate the burden of influenza and understand the effect on populations.

While most studies assessed the impact of influenza on circulatory deaths in term of cardiorespiratory deaths,^13^, ^21^ we evaluated it separately. We found that the impact of influenza on circulatory deaths was similar to that of respiratory deaths (3.6 vs. 3.4 excess rate per 100,000 population, respectively). This would translate to an annual excess of 2877 respiratory and 2717 circulatory deaths for the whole country. However, we should interpret these figures with caution, because we observed that 8.9% of all respiratory and 2.4% of all circulatory deaths were attributed to influenza. This highlights the greater impact of influenza on respiratory deaths in Iran.

Older adults showed the highest excess mortality associated with influenza. We found that adults ≥75 years accounted for 56% and 53% of all excess respiratory and circulatory deaths, respectively. Our findings are in accord with WHO recommendations for vaccination of older adults to reduce the mortality burden of seasonal influenza.^28^ In Iran, influenza vaccination is only distributed by MOHME.^15^, ^29^ Vaccines are available free of charge for vaccine‐recommended groups (including pregnant women, children 6–59 months of age, adults aged ≥60 years, health care workers, persons with chronic illness, and poultry workers) through the public health sector. However, individuals not in these recommended groups have to buy the vaccine by themselves own through the private health sector.^29^ The country's vaccination coverage is unknown^15^, ^29^; however, studies show a varied picture of vaccination coverage/history among different populations. The vaccination coverage among people living with HIV/AIDS and health care providers at triangular clinics, which offer HIV care and harm reduction services, were 56% and 74%, respectively.^30^ Receipt of influenza vaccine in the past year was reported as 2% among patients diagnosed with acute lower respiratory tract infection,^31^ 51% among physicians and nurses,^32^ and 100% among Hajj Pilgrims.^33^, ^34^ It seems that increasing awareness of the benefit of vaccination for seasonal influenza in Iran is needed.

Studies suggest that there is no gold standard modeling approach to estimate influenza‐associated deaths.^6^, ^35^ As our dependent variable was the count of deaths in a given week and might be over‐dispersed, we tried to assess the impact of over‐dispersion by introducing the Serfling approach in a negative binomial model. Although we have found a strong association between the results of Serfling linear and negative binomial models and a similar baseline predicted mortality in the absence of influenza activity, future studies should be conducted to confirm our results with and without inclusion of influenza viral surveillance parameters in statistical modeling approaches.

There are some considerations in interpreting of our findings. Although the completeness of the death registry system in Kerman is the highest in Iran,^36^ there is no information on accuracy of cause of death coding practice over the country, including Kerman. As our results are dependent on the accuracy of death coding, which might differ in different locations, we must assume that it was consistent and accurate over the province during the study period. Regardless of the accuracy of death coding practice, the Iran's MOHME tries to inspect the quality of death records by removing duplicates from different locations, checking the ICD‐10 codes with plausible age and/or sex and asking medical universities to correct infeasibilities, correcting missing information on sex/age through redistribution methods, correcting non‐fatal health conditions based on global burden of disease framework, and distributing ill‐defined and unusable codes in age‐sex grouping, regularly.^37^ Estimating all‐cause deaths associated with influenza can indicate the total burden of influenza in the population, which may overestimate the true burden by attributing deaths due to injuries or violence to influenza. Furthermore, our estimates for the country must be interpreted while considering assumptions that influenza periods are similar in timing and duration between Kerman and other provinces.

Our study has some limitations that should be addressed. Although we found variations over the six influenza seasons, we could not attribute these variations to influenza virus types/subtypes. The influenza viral surveillance system was not robust and validated at that time; there was only one reference laboratory that tested all viral specimens across the country, the National Influenza Center (NIC) at Tehran University of Medical Sciences. However, there were no records for Kerman province in surveillance records during the study period. The Kerman sub‐NIC was established in January 2013. We believe that future studies with more robust viral data could provide estimates of the mortality burden of influenza by virus type/subtype. Because, like Iran, Kerman province has a diverse climate (including both cold and warm regions), the start and length of epidemic periods may vary among provinces. This might be one source of variability on our mortality data that would need to be accounted in future studies. We did not assess the mortality impact of the influenza A(H1N1)pdm09 pandemic, in part because the observed PI death rates were nearly stable in different seasons. However, the mortality rate of A(H1N1)pdm09 in Iran was previously estimated at 2 per 1,000,000 persons.^38^

## 5 CONCLUSION

In Kerman province, we found a mortality burden of seasonal influenza similar to that of other studies and a disproportionate burden among adults aged ≥75 years. Our findings highlight the potential value of comprehensive influenza prevention strategies, including vaccination programs in older adults, campaigns for awareness of influenza vaccination, and simple prevention strategies (i.e., hand washing and respiratory hygiene).

## AUTHOR CONTRIBUTIONS

**Razieh Khajehkazemi:** Conceptualization; methodology; data curation; formal analysis; validation; writing‐original draft; writing‐review and editing. **Mohammad Reza Baneshi:** Conceptualization; methodology; formal analysis; validation; writing‐review and editing. **Angela Danielle Iuliano:** Conceptualization; methodology; formal analysis; validation; writing‐review and editing. **Katherine M. Roguski:** Methodology; formal analysis; validation; writing‐review and editing. **Hamid Sharifi:** Methodology; validation; writing‐review and editing. **Joseph Bresee:** Conceptualization; validation; writing‐review and editing. **AliAkbar Haghdoost:** Conceptualization; conceptualization; methodology; data curation; validation; formal analysis; funding acquisition; supervision; writing‐review and editing.

## CONFLICT OF INTEREST

No conflict of interest declared.

## ETHICS APPROVAL

The ethics committee of the Kerman University of Medical Sciences approved our study (code IR.KMU.REC.1395.423).

## PATIENT CONSENT TO PARTICIPATE

Because we used aggregated data and there was no personal information, no informed consent was required.

## DISCLAIMER

The findings and conclusions in this report are those of the authors and do not necessarily represent the official position of the US Centers for Disease Control and Prevention.

### PEER REVIEW

The peer review history for this article is available at https://publons.com/publon/10.1111/irv.13061.

## Supporting information


**Table S1:** Mortality rate per 100,00 population by age groups and underlying cause of death, influenza seasons 2006/2007‐2011/2012, Kerman province, Iran
**Table S2:** Epidemic weeks and duration of epidemic periods defined by Serfling linear and negative binomial regression models during annual influenza seasons 2006/2007 to 2011/2012, Kerman province, Iran
**Table S3:** Sensitivity Analysis: Estimated influenza‐associated mortality by age groups and underlying cause of death, during annual influenza seasons 2006/2007 to 2011/2012, Kerman province, Iran using Serfling negative binomial model
**Figure S1:** Observed weekly respiratory mortality rates per 100,000 population and baseline predicted rates fitted by Serfling linear and negative binomial models among people aged ≥75 years
**Figure S2:** Observed weekly circulatory mortality rates per 100,000 population and baseline predicted rates fitted by Serfling linear and negative binomial models among people aged ≥75 years
**Figure S3:** Observed weekly all‐cause mortality rates per 100,000 population and baseline predicted rates fitted by Serfling linear and negative binomial models among people aged ≥75 years
**Text S1:** Study location and the death certificate and registry process in Iran.

## Data Availability

The data that support the findings of this study are available from Ministry of Health and Medical Education of Iran but restrictions apply to the availability of these data, which were used under license for the current study, and so are not publicly available.
