# Supplementary material for: Estimated mortality due to seasonal influenza in southeast of Iran, 2006/2007 to 2011/2012 influenza seasons
Source: Influenza Other Respir Viruses. 2022 Oct 26;17(1):e13061. doi: 10.1111/irv.13061 (PMC9835411; doi:10.1111/irv.13061)
Supplement: Supplementary file 1 — Table S1: Mortality rate per 100,00 population by age groups and underlying cause of death, influenza seasons 2006/2007‐2011/2012, Kerman province, Iran Table S2: Epidemic weeks and duration of epidemic periods defined by Serfling linear and negative binomial regression models during annual influenza seasons 2006/2007 to 2011/2012, Kerman province, Iran Table S3: Sensitivity Analysis: Estimated influenza‐associated mortality by age groups and underlying cause of death, during annual influenza seasons 2006/2007 to 2011/2012, Kerman province, Iran using Serfling negative binomial model Figure S1: Observed weekly respiratory mortality rates per 100,000 population and baseline predicted rates fitted by Serfling linear and negative binomial models among people aged ≥75 years Figure S2: Observed weekly circulatory mortality rates per 100,000 population and baseline predicted rates fitted by Serfling linear and negative binomial models among people aged ≥75 years Figure S3: Observed weekly all‐cause mortality rates per 100,000 population and baseline predicted rates fitted by Serfling linear and negative binomial models among people aged ≥75 years Text S1: Study location and the death certificate and registry process in Iran. [file IRV-17-e13061-s001.docx]

**Supplementary information**

**Text S1.** Study location and the death certificate and registry process in Iran.

**Study location**

Kerman province is located in latitudes 26.29⁰ (i.e. Ghale Ganj county) to 31.58⁰ (i.e. Ravar county) North and longitudes 54.20⁰ to 59.34⁰ East^1^. The province has a varying climate. The mean maximum monthly temperature ranges from 12 to 30⁰C (53.6 to 86⁰F) in cooler months (October-May) and 31 to 36⁰C (87.7 to 96.8⁰F) in warmer moths (June-September); the corresponding values for the mean minimum monthly temperature are -4 to 12⁰C (24.8 to 53.6⁰F) and 10 to 17⁰C (50 to 62.6⁰F), respectively^2^.

**Death certificate process in Iran**

Death certificate is completed and issued by a qualified physician/midwife, who successfully passed training courses on “properly completing and filling death certificates” and received a certificate of completion. These training courses are held by medical universities or forensic medicine bureaus. Midwives are only authorized to issue death certificate for stillbirths and neonatal deaths less than seven days of age. In general, the completion of death certificate in Iran can be classified in three major categories: (i) for deaths occur at hospitals (where more than 60% of deaths occur) and maternity hospitals and natural deaths occur out of hospitals (e.g. home, private and public clinics), the physician approves the death and issues the death certificate, (ii) for suspicious and abnormal deaths and deaths occur at residential centers (e.g. prisons and dormitories), a physician at forensic medicine bureau determines the exact cause of death and issues the death certificate, (iii) for natural deaths occur at home in villages and rural areas in case of unavailability of a physician, the verbal autopsy form is filled by a trained health care worker at rural health center^3-6^.

**Death registration process**

Death certificate is issued in three copies. The district health center received one copy of death certificate from various sources including hospitals, maternity hospitals, urban and rural health centers, private and public clinics, forensic medicine bureaus, and cemeteries located in that district; and evaluated it qualitatively for missing/insufficient information and unlikely causes of death; and quantitatively to remove duplicates and find missed death certificates for those deaths reported by Civil Registration Bureau but the form was not received from the above sources. The death certificate will be returned to the person who signed it, if it is incomplete.

A trained person in district health center, who had a medical records degree or passed training courses on “how to determine the appropriate and assign an ICD-10 code” and “how to use the death registry software”, registers the deceased information into the death registry software and assigns the ICD-10 codes for immediate, antecedent, and underlying causes of death. Then, the information is uploaded to the online integrated national death registry system. The data is checked to remove duplicates between different districts by the university’s Vice-Chancellor for Health. Then, the Department of Statistics and Informatics, Network Management Center, Health Deputy, Ministry of Health and Medical Education of Iran will check the data to remove duplicates between different universities and do some corrections (more information can be found in the discussion)^3-6^.

**References**

1. Borjian H. Keman i. Geography. Encyclopaedia Iranica <http://www.iranicaonline.org/articles/kerman-geography> Accessed Aug 16, 2020
2. Weather Atlas. Detailed climate information and monthly weather forecast. Kerman, Iran- Average monthly weather. <https://www.weather-atlas.com/en/iran/kerman-climate#temperature> Accessed Aug 1, 2019
3. Khosravi A, Aghamohamadi S, Kazemi. Guideline on death registry and classification of cause of death. Tehran, Iran: Ministry of Health and Medical Education; 2015.
4. Jafari N, Kabir MJ, Motlagh ME. Death registration system in IR Iran. Iranian Journal of Public Health. 2009;38(1):127-129.
5. Khosravi A, Rao C, Naghavi M, Taylor R, Jafari N, Lopez AD. Impact of misclassification on measures of cardiovascular disease mortality in the Islamic Republic of Iran: a cross-sectional study. Bulletin of the World Health Organization. 2008;86:688-696.
6. Sheidaei A, Gohari K, Kasaeian A, Rezaei N, Mansouri A, Khosravi A, Parsaeian M, Mohammadi Y, Mehdipour P, Rahimzadeh S, Sharifi F, Varmaghani M, Chegini M, Naderimagham S, Hamshidi HR, Farzadfar F.National and subnational patterns of cause of death in Iran 1990-2015: Applied methods. Archives of Iranian medicine. 2017;20(1):2-11.

**Table S1:** Mortality rate per 100,00 population by age groups and underlying cause of death, influenza seasons 2006/2007-2011/2012, Kerman province, Iran

| **Underlying cause of death and Seasons** | **Age groups (years)** | | | |
| --- | --- | --- | --- | --- |
|  | **≤64** | **65-74** | **≥75** |  |
|  | **Rate (% of total†)** | **Rate (% of total†)** | **Rate (% of total†)** | **All ages** |
| **PI** |  |  |  |  |
| 2006/2007 | 2.9 (32.7) | 53.5 (17.7) | 222.0 (49.6) | 8.5 |
| 2007/2008 | 3.1 (32.8) | 56.4 (17.6) | 230.2 (49.6) | 9.0 |
| 2008/2009 | 1.9 (28.2) | 30.9 (13.6) | 188.3 (58.2) | 6.4 |
| 2009/2010 | 2.9 (37.4) | 32.8 (12.3) | 186.6 (50.2) | 7.5 |
| 2010/2011 | 2.3 (33.9) | 35.9 (15.3) | 162.8 (50.8) | 6.6 |
| 2011/2012 | 1.8 (24.9) | 30.4 (12.2) | 211.2 (62.9) | 7.0 |
| **Respiratory** |  |  |  |  |
| 2006/2007 | 11.6 (26.7) | 340.0 (22.9) | 1104.0 (50.3) | 41.5 |
| 2007/2008 | 11.7 (26.8) | 254.6 (17.1) | 1207.9 (56.1) | 41.6 |
| 2008/2009 | 10.9 (28.4) | 248.3 (19.1) | 971.0 (52.5) | 36.5 |
| 2009/2010 | 13.3 (32.9) | 237.2 (17.4) | 948.6 (49.8) | 38.4 |
| 2010/2011 | 14.2 (32.6) | 250.1 (17.0) | 1014.3 (50.4) | 41.3 |
| 2011/2012 | 10.8 (25.3) | 224.9 (15.6) | 1146.2 (59.1) | 40.5 |
| **Circulatory** |  |  |  |  |
| 2006/2007 | 42.4 (28.2) | 1203.4 (23.5) | 3670.6 (48.4) | 143.4 |
| 2007/2008 | 37.0 (25.2) | 1119.3 (22.5) | 3777.8 (52.3) | 139.6 |
| 2008/2009 | 38.0 (25.9) | 1057.6 (21.2) | 3752.3 (52.9) | 139.9 |
| 2009/2010 | 43.9 (27.2) | 1052.5 (19.3) | 4075.9 (53.5) | 153.3 |
| 2010/2011 | 40.8 (26.4) | 958.3 (18.3) | 3970.7 (55.3) | 147.1 |
| 2011/2012 | 37.2 (27.5) | 788.8 (17.2) | 3415.6 (55.3) | 128.8 |
| **All cause** |  |  |  |  |
| 2006/2007 | 215.6 (50.2) | 2346.6 (16.0) | 7309.5 (33.7) | 409.3 |
| 2007/2008 | 202.3 (48.6) | 2199.1 (15.5) | 7365.4 (35.9) | 396.6 |
| 2008/2009 | 193.5 (48.6) | 2084.4 (15.4) | 6910.4 (36.0) | 379.0 |
| 2009/2010 | 204.2 (49.1) | 2006.5 (14.2) | 7231.4 (36.7) | 396.2 |
| 2010/2011 | 206.8 (49.2) | 1899.3 (13.3) | 7308.7 (37.5) | 400.0 |
| 2011/2012 | 188.8 (48.8) | 1719.9 (13.2) | 6695.3 (38.0) | 367.6 |
| **PI= pneumonia and influenza**  **† Calculated as the percentage of deaths in each age and cause group in a given season among total deaths of that cause in that season.** | | | | |

**Table S2:** Epidemic weeks and duration of epidemic periods defined by Serfling linear and negative binomial regression models during annual influenza seasons 2006/2007 to 2011/2012, Kerman province, Iran

| **Seasons** | **Serfling linear model** | | | **Serfling negative binomial model** | | |
| --- | --- | --- | --- | --- | --- | --- |
|  | **Epidemic weeks** | **Epidemic periods** | **Epidemic period length** | **Epidemic weeks** | **Epidemic periods** | **Epidemic period length** |
| 2006/2007 | 40, 49, 52,1,5-6,9,11,13-15,17,19 | 52-1, 5-19 | 17 | 40,49,52,5-6,9,11,13-15,17,19 | 5-19 | 15 |
| 2007/2008 | 42,46-47,50,1,3-5,7,11-14,16-18,20 | 46-7, 11-20 | 24 | 42,46-47,50,1,3-4,7,11-14,16-18,20 | 46-7, 11-20 | 24 |
| 2008/2009 | 50-51,4,6,9,14 | 50-51, 4-9 | 8 | 50-51,4,6,9,14 | 50-51, 4-9 | 8 |
| 2009/2010 | 41-46,50,52,12,16,19 | 41-46, 50-52 | 9 | 41-46,50,52,12,16,19 | 41-46, 50-52 | 9 |
| 2010/2011 | 43,48,50,3,6,8,10 | 48-50, 6-10 | 8 | 43,48,50,3,6,8,10 | 48-50, 6-10 | 8 |
| 2011/2012 | 40,42,46-47,50-52,3-5,8,12-15 | 40-42, 46-8, 12-15 | 22 | 40,42,46-47,50-52,3-5,8,12-15 | 40-42, 46-8, 12-15 | 22 |

**Table S3:** Sensitivity Analysis: Estimated influenza-associated mortality by age groups and underlying cause of death, during annual influenza seasons 2006/2007 to 2011/2012, Kerman province, Iran using Serfling negative binomial model

| **Underlying cause of death and seasons** | **Excess death number** | | | | **Excess death rate per 100,000 population (95% CI)** | | | |
| --- | --- | --- | --- | --- | --- | --- | --- | --- |
|  | **≤64** | **65-74** | **≥75** | **Total** | **≤64** | **65-74** | **≥75** | **Total** |
| **PI** |  |  |  |  |  |  |  |  |
| 2006/2007 | NA | NA | NA | 38 | NA | NA | NA | 1.4 (1.1-1.8) |
| 2007/2008 | NA | NA | NA | 59 | NA | NA | NA | 2.2 (1.6-2.7) |
| 2008/2009 | NA | NA | NA | 12 | NA | NA | NA | 0.4 (0.3-0.6) |
| 2009/2010 | NA | NA | NA | 35 | NA | NA | NA | 1.2 (1.1-1.4) |
| 2010/2011 | NA | NA | NA | 18 | NA | NA | NA | 0.6 (0.4-0.8) |
| 2011/2012 | NA | NA | NA | 72 | NA | NA | NA | 2.5 (2-3) |
| Mean | NA | NA | NA | 39 | NA | NA | NA | 1.4 (1.1-1.7) |
| **Respiratory** |  |  |  |  |  |  |  |  |
| 2006/2007 | 36 | 31 | 37 | 104 | 1.4 (1-1.9) | 41.9 (28.2-59.1) | 72.4 (47.3-100.9) | 3.9 (2.7-5.4) |
| 2007/2008 | 32 | 23 | 83 | 138 | 1.3 (0.7-1.9) | 30.7 (20.7-46) | 157.6 (124.8-205.8) | 5.1 (3.7-7.1) |
| 2008/2009 | 16 | 4 | 19 | 39 | 0.6 (0.4-0.8) | 5.1 (3.9-6.3) | 35.2 (27.2-45.4) | 1.4 (1.1-1.9) |
| 2009/2010 | 19 | 11 | 18 | 48 | 0.7 (0.6-0.9) | 14.2 (10.8-18.4) | 31 (20-44) | 1.7 (1.2-2.3) |
| 2010/2011 | 10 | 5 | 21 | 36 | 0.4 (0.2-0.6) | 5.7 (2.3-9.8) | 35.2 (21.7-53.3) | 1.2 (0.8-1.9) |
| 2011/2012 | 33 | 32 | 147 | 212 | 1.2 (0.7-1.7) | 39 (27.8-55.2) | 240 (189.3-295.3) | 7.3 (5.5-9.3) |
| Mean | 25 | 18 | 54 | 96 | 0.9 (0.6-1.3) | 22.8 (15.6-32.5) | 95.2 (71.7-124.1) | 3.4 (2.5-4.7) |
| **Circulatory** |  |  |  |  |  |  |  |  |
| 2006/2007 | 29 | 9 | 27 | 65 | 1.1 (0.6-2) | 12 (3.6-25.6) | 52.8 (23.7-82.8) | 2.4 (1.1-4.2) |
| 2007/2008 | 25 | 52 | 111 | 188 | 1 (0.6-1.6) | 68.5 (47.7-98.7) | 208.7 (160-270) | 6.9 (5.0-9.6) |
| 2008/2009 | 2 | 13 | 25 | 40 | 0.1 (0-0.3) | 16.3 (5.7-32.2) | 45.3 (25.4-69.5) | 1.4 (0.6-2.6) |
| 2009/2010 | 42 | 17 | 41 | 100 | 1.6 (1.3-1.9) | 21.6 (13.1-32.4) | 73.1 (54.6-101.1) | 3.6 (2.7-4.8) |
| 2010/2011 | 13 | 9 | 31 | 53 | 0.5 (0.3-0.8) | 11.4 (5.4-18.2) | 53 (28.9-86) | 1.8 (1.0-3.1) |
| 2011/2012 | 14 | 36 | 66 | 116 | 0.5 (0.1-1.2) | 43.8 (29-72.1) | 107.8 (63.7-178.7) | 4.0 (2.3-6.9) |
| Mean | 21 | 23 | 50 | 94 | 0.8 (0.5-1.3) | 28.9 (17.4-46.5) | 90.1 (59.4-131.3) | 3.4 (2.1-5.2) |
| **All Cause** |  |  |  |  |  |  |  |  |
| 2006/2007 | 97 | 33 | 65 | 195 | 3.8 (2.1-6.8) | 44.2 (18-86) | 127.6 (53.4-213) | 7.3 (3.6-13.0) |
| 2007/2008 | 300 | 96 | 241 | 637 | 11.5 (8.9-14.6) | 126.1 (92.3-166.8) | 455 (360-557.7) | 23.5 (18.2-29.5) |
| 2008/2009 | 81 | 36 | 61 | 178 | 3.1 (2-4.6) | 46.7 (35.8-60.1) | 111.9 (65.7-163.7) | 6.4 (4.2-9.4) |
| 2009/2010 | 71 | 28 | 36 | 135 | 2.6 (1.7-4) | 36 (24.3-49.4) | 64.7 (48.3-98.6) | 4.8 (3.2-7.2) |
| 2010/2011 | 94 | 17 | 74 | 185 | 3.4 (2-5.2) | 21.1 (14.7-31.7) | 125.2 (82.4-182.6) | 6.4 (4.1-9.6) |
| 2011/2012 | 186 | 88 | 257 | 531 | 6.7 (3.9-10.1) | 106.8 (70.9-153) | 420.5 (281-572.1) | 18.2 (11.6-25.9) |
| Mean | 138 | 50 | 122 | 310 | 5.2 (3.4-7.5) | 63.5 (42.7-91.2) | 217.5 (148.5-297.9) | 11.1 (7.5-15.8) |
| **NA= not applicable (due to excess zeros in the age-specific PI mortality data); PI= pneumonia and influenza; CI= confidence interval** | | | | | | | | |


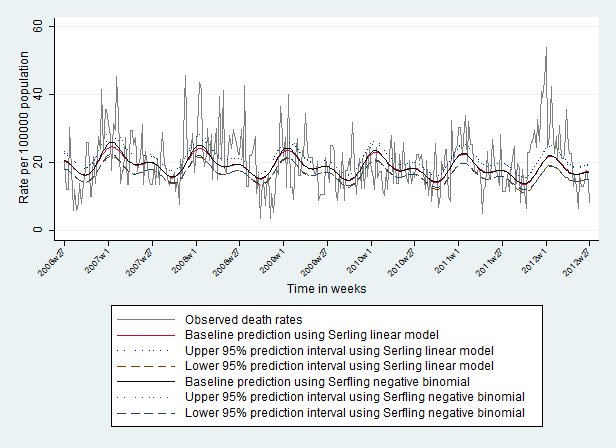


**Figure S1:** Observed weekly respiratory mortality rates per 100,000 population and baseline predicted rates fitted by Serfling linear and negative binomial models among people aged ≥75 years


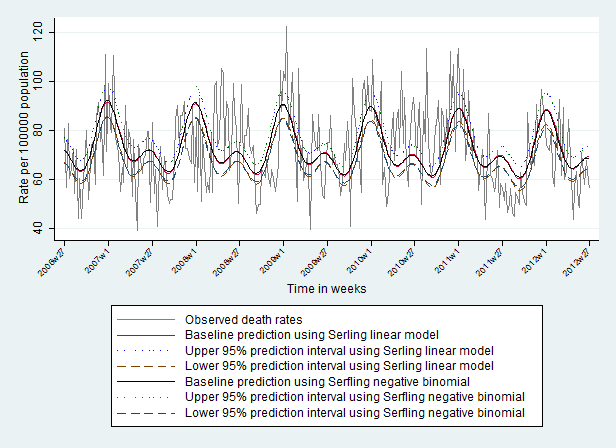


**Figure S2:** Observed weekly circulatory mortality rates per 100,000 population and baseline predicted rates fitted by Serfling linear and negative binomial models among people aged ≥75 years


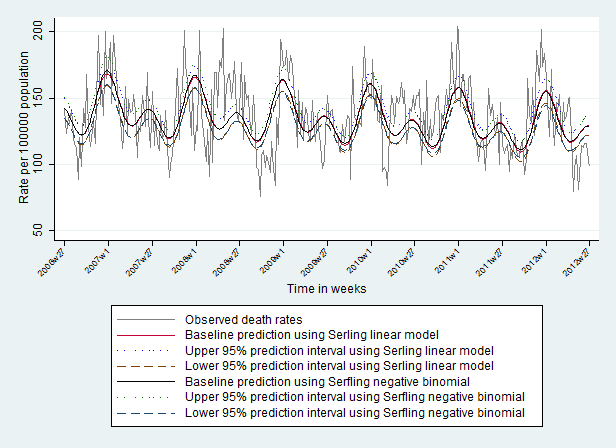


**Figure S3:**Observed weekly all-cause mortality rates per 100,000 population and baseline predicted rates fitted by Serfling linear and negative binomial models among people aged ≥75 years
